# Supplementary material for: The Cancer-Associated Fibroblasts-Related Gene COMP Is a Novel Predictor for Prognosis and Immunotherapy Efficacy and Is Correlated with M2 Macrophage Infiltration in Colon Cancer
Source: Biomolecules. 2022 Dec 28;13(1):62. doi: 10.3390/biom13010062 (PMC9856124; doi:10.3390/biom13010062)
Supplement: Supplementary file 1 [file biomolecules-13-00062-s001.zip › FIgure captions.pdf]

Figure S1: Assessment of clinical features and the immune landscape asso- 594 ciated with CAFs in the TCGA and GSE17536 cohort. (A) Correlations of different TME cell types 595 with stromal, immune and ESTIMATE scores as well as tumor purity in the TCGA cohort. (B) Var- 596 ious TME cell abundances in the GSE17436 cohort were shown in the heat map. (B) (C) Correlations 597 of different TME cell types with stromal, immune and ESTIMATE scores as well as tumor purity in 598 the GSE17536 cohort;

Figure S2: The network topology in WGCNA. (A) and (B) show the soft thresh- 599 olds in the TCGA and GSE17536 cohort, respectively;

Figure S3: COMP-related biological processes 600 were enriched by (A) GO and (B) KEGG analyses;

Figure S4: K-M plots for high- and low-COMP 601 expression subgroups of (A) TCGA, (B) GSE17536, (C) GSE39582, (D) GSE41258 and (E) the com- 602 bined-GEO cohorts;

Figure S5: Assessment of the 22 immune cell fractions in the low- and high- 603 COMP expression subgroups by the CIBERSORT algorithm. (A) Association between CAFs, stromal 604 score, immune score, tumor purity, and COMP expression. (B) Different infiltrating patterns of 22 605 immune cells across different COMP subgroups in TCGA and GSE17536. (B) Estimation of the 22 606 immune cell fractions in the low- and high-COMP expression subgroups in the (C) TCGA and (D) 607 GSE17536 datasets;.

Figure S6: Correlation of COMP with somatic mutations. (A) The landscape of 608 somatic mutations showed the top 15 genes exhibiting the highest mutation frequency. (B) Twelve 609 genes more frequently mutated in the high-COMP subgroup were input in GSCA database as a 610 gene set, and the association between the abundance of naïve CD8+ T cells, CD8+ T cells, macro- 611 phages and the gene set mutation level was exhibited. (C) The correlation between COMP expres- 612 sion and TMB. (D) COMP expression in patients with MSS/MSI-L and MSI-H;

Figure S7: The correlations between COMP expression and putative immunotherapy response in external cohorts. The corre- 613 lations between COMP expression and the TIDE score, the proportions of patients with putative 614 immunotherapy response in two COMP expression subgroups, and the comparisons of COMP ex- 615 pression between distinct responses in the (A) GSE17536, (B) GSE39582 and (C) GSE41258 cohorts. 616 R, responder; NR, non-responder. (D) Evaluation of COMP expression in anti-PD-L1 responders 617 versus non-responders and ROC curves of COMP expression in the IMvigor210 cohort. (E) K–M 618 plots for aRCC patients treated with the combination of avelumab and axitinib versus sunitinib 619 alone in the whole JAVELIN Renal 101 cohort.
