# Supplementary figures and images for: The Cancer-Associated Fibroblasts-Related Gene COMP Is a Novel Predictor for Prognosis and Immunotherapy Efficacy and Is Correlated with M2 Macrophage Infiltration in Colon Cancer
Source: Biomolecules. 2022 Dec 28;13(1):62. doi: 10.3390/biom13010062 (PMC9856124; doi:10.3390/biom13010062)

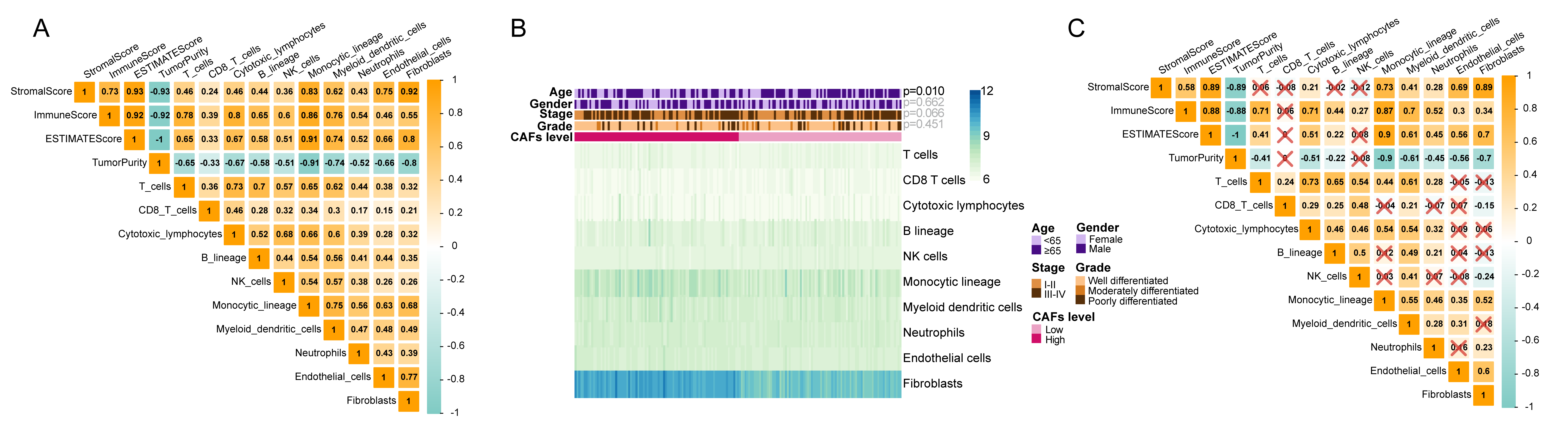

Supplement: Supplementary file 1 [file biomolecules-13-00062-s001.zip › Figure S1.tif]

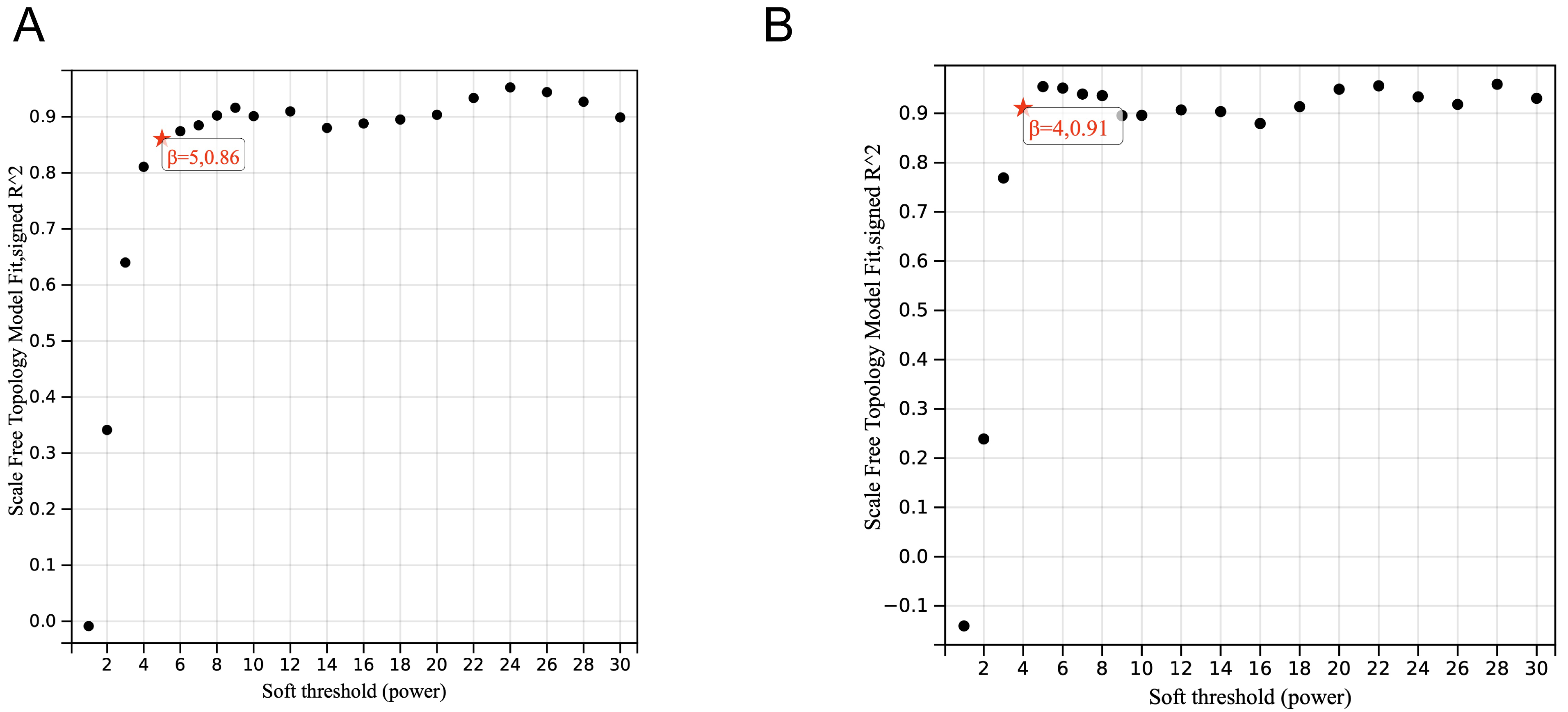

Supplement: Supplementary file 1 [file biomolecules-13-00062-s001.zip › Figure S2.tif]

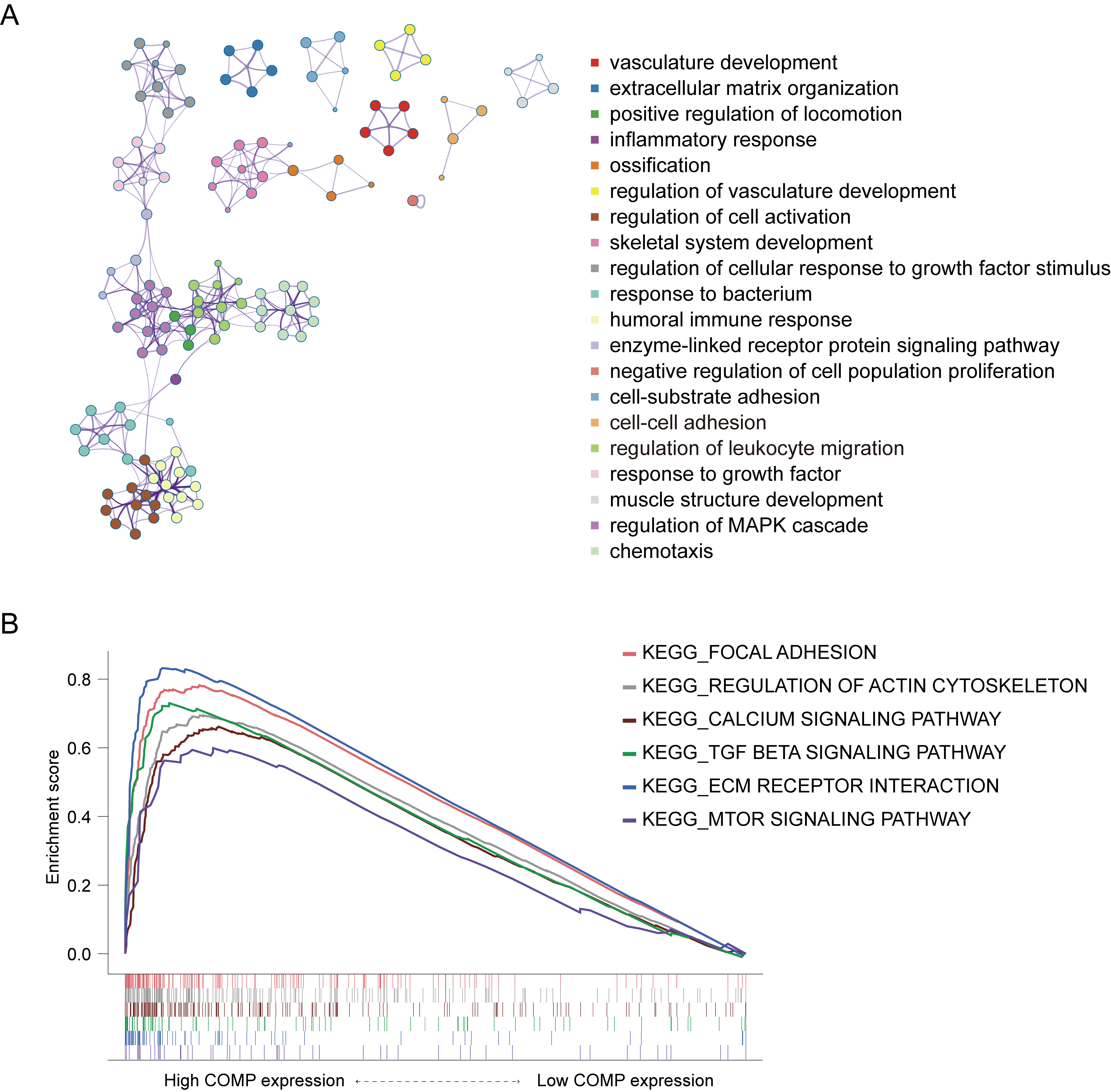

Supplement: Supplementary file 1 [file biomolecules-13-00062-s001.zip › Figure S3.tif]

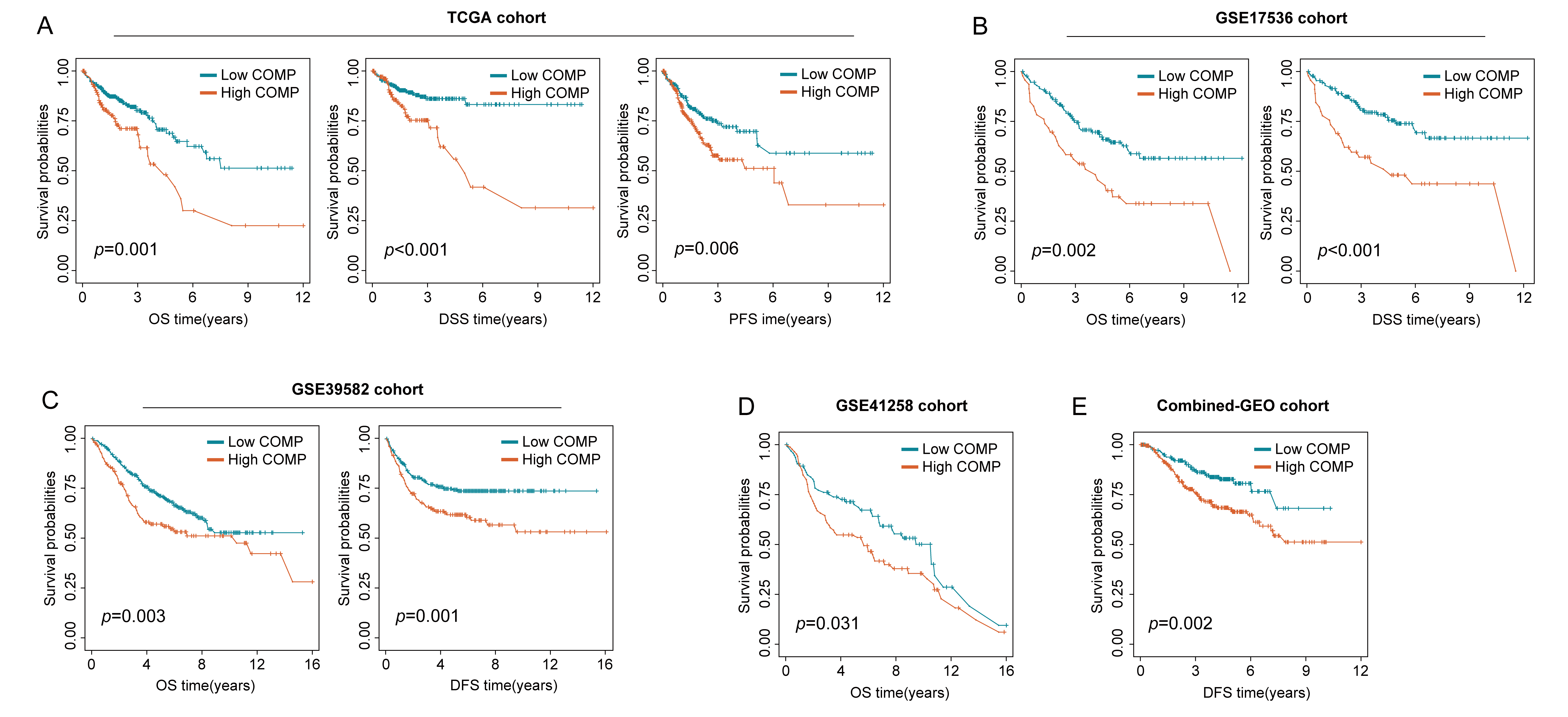

Supplement: Supplementary file 1 [file biomolecules-13-00062-s001.zip › Figure S4.tif]

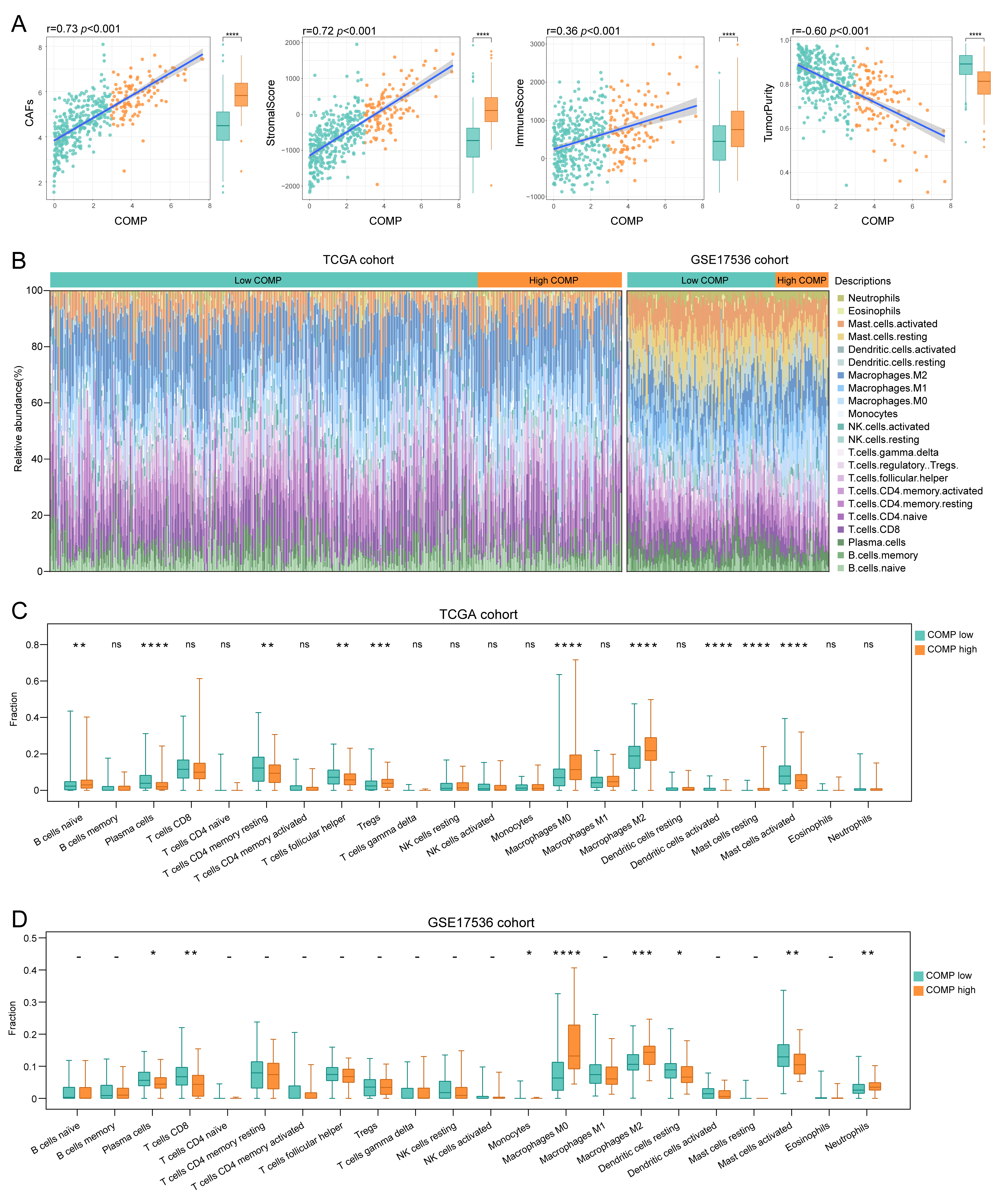

Supplement: Supplementary file 1 [file biomolecules-13-00062-s001.zip › Figure S5.tif]

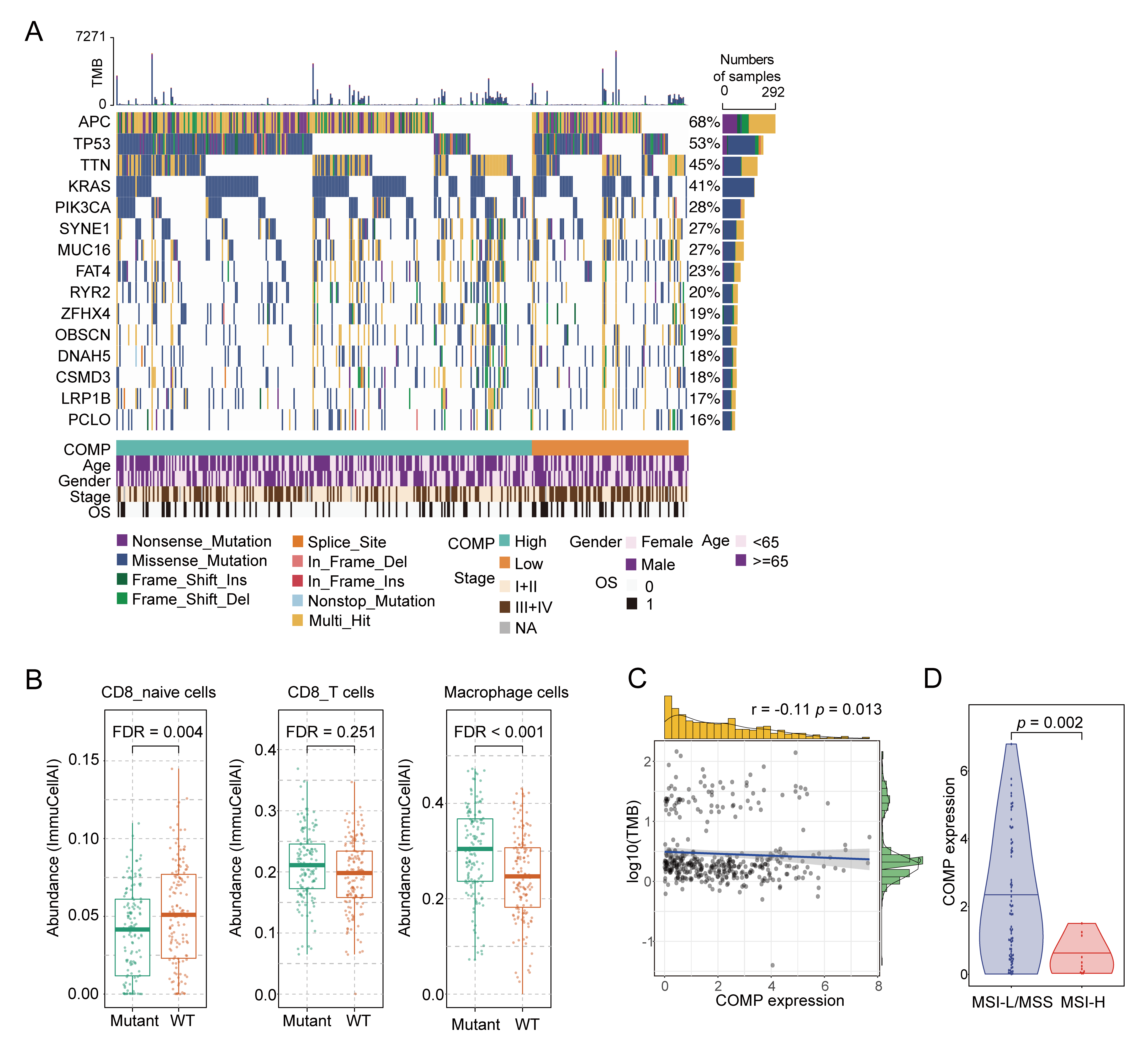

Supplement: Supplementary file 1 [file biomolecules-13-00062-s001.zip › Figure S6.tif]

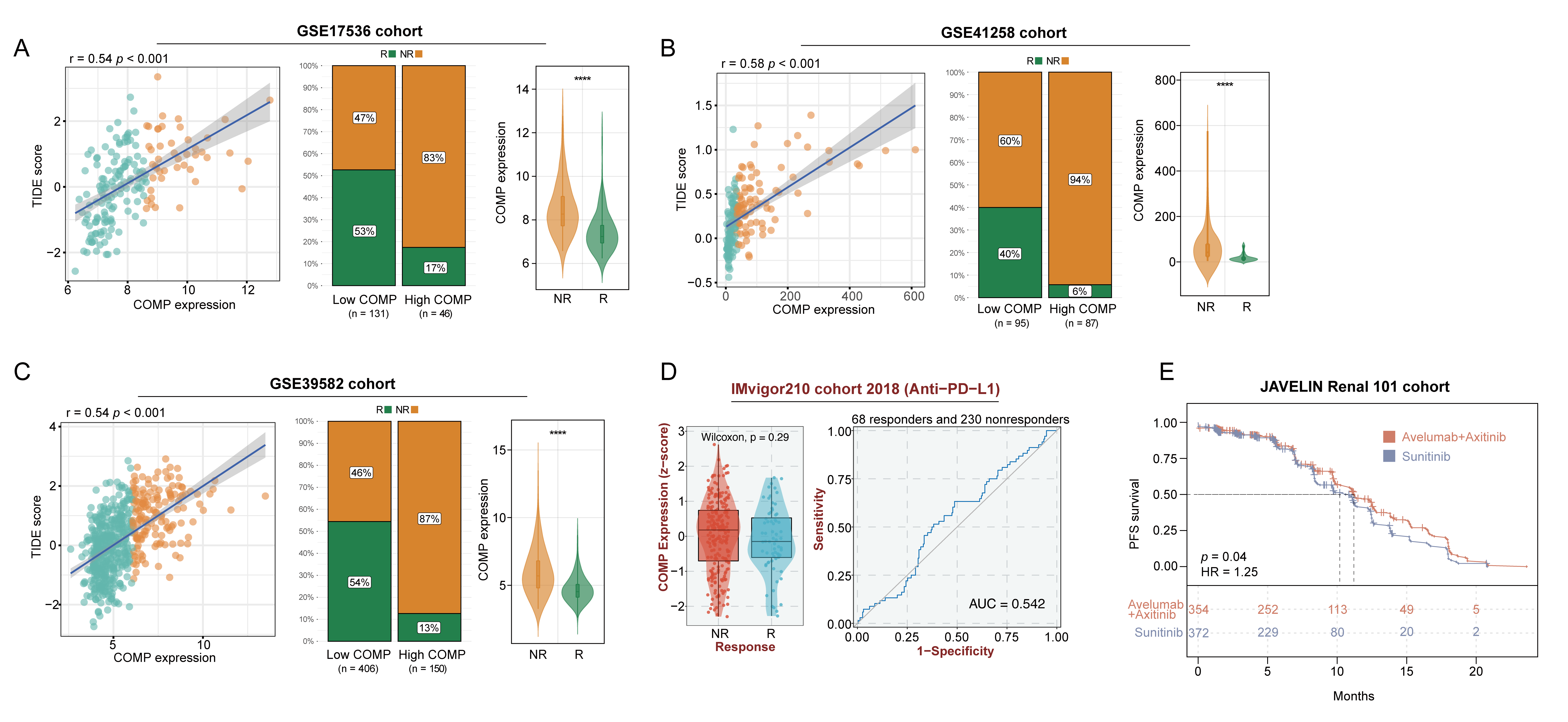

Supplement: Supplementary file 1 [file biomolecules-13-00062-s001.zip › Figure S7.tif]
